# Perceived risk of COVID-19 hurts mental health: the mediating role of fear of COVID-19 and the moderating role of resilience

**DOI:** 10.1186/s12888-024-05511-x

**Published:** 2024-01-22

**Authors:** Hui Lu, Jialin Yang, Kejie Zhao, Zhou Jin, Xin Wen, Nuonuo Hu, Hongshen Yang, Zhiyu Sun, Haitao Chen, Yili Huang, Deborah Baofeng Wang, Yili Wu

**Affiliations:** 1 Oujiang Laboratory (Zhejiang Lab for Regenerative Medicine, Vision and Brain Health), Key Laboratory of Alzheimer’s Disease of Zhejiang Province, Wenzhou Key Laboratory of Basic and Translational Research for Mental Disorders, School of Mental Health and The Affiliated Wenzhou Kangning Hospital, Institute of Aging, Zhejiang Provincial Clinical Research Center for Mental Disorders, Wenzhou Medical University, Wenzhou, Zhejiang 325000 China; 2 Zhejiang Provincial Clinical Research Center for Mental Disorders, Wenzhou Key Laboratory of Basic and Translational Research for Mental Disorders, School of Mental Health and The Affiliated Wenzhou Kangning Hospital, Key Laboratory of Alzheimer’s Disease of Zhejiang Province, Institute of Aging, Oujiang Laboratory (Zhejiang Lab for Regenerative Medicine, Vision and Brain Health), Wenzhou Medical University, Wenzhou, Zhejiang 32500 China; 3 Lyons Insights Consulting, Chicago, United States of America

**Keywords:** Depression, Anxiety, COVID-19 perceived risk, Fear of COVID-19, Resilience

## Abstract

**Background:**

Depression and anxiety have been found prevalent during all phases of the COVID-19 pandemic. In late December 2022, almost all COVID-19 control measures were lifted in China, leading to a surge in COVID-19 infections. The public’s perceived risk and fear of COVID-19 would be increased. This study aims to examine the prevalence of depression and anxiety in the Chinese general population and explores the mediating role of fear of COVID-19 between COVID-19 perceived risk and depression/anxiety and the moderating role of resilience between fear of COVID-19 and depression/anxiety.

**Methods:**

A cross-sectional online survey was conducted in Wenzhou, China, immediately following almost all COVID-19 control measures lifted. The 9-item Patient Health Questionnaire (PHQ-9), Generalized Anxiety Disorder-7 (GAD-7), the COVID-19 Risk Perception Scale, the Fear of COVID-19 Scale, and the Connor-Davidson Resilience Scale (CD-RISC) were used to evaluate depression, anxiety, COVID-19 perceived risk, fear of COVID-19, and resilience, respectively. Structural Equation Modeling (SEM) with Maximum Likelihood (ML) estimator and adjusted for significant background factors was performed to test the moderated mediation. Data obtained from 935 participants were analyzed.

**Results:**

The prevalence of moderate to severe depression and anxiety was 23.7% and 9.5%, respectively. The present study revealed positive associations among COVID-19 perceived risk, fear of COVID-19 and depression/anxiety, and negative associations between resilience and fear of COVID-19/depression/anxiety. Fear of COVID-19 partially mediated the association between COVID-19 perceived risk and depression/anxiety. Furthermore, resilience significantly moderated the association between fear of COVID-19 and depression/anxiety. Two moderated mediation models were constructed.

**Conclusion:**

Depression and anxiety were prevalent among Chinese adults during the final phase of the pandemic in China. The significant mediation role of fear of COVID-19 implies that reducing fear of COVID-19 may effectively alleviate depression and anxiety symptoms. Moreover, enhancing public resilience during an epidemic crisis is crucial for promoting mental health.

**Supplementary Information:**

The online version contains supplementary material available at 10.1186/s12888-024-05511-x.

## Introduction

Depression and anxiety are prevalent mental distress that individuals commonly experience during the pandemic and the levels of distress may be dramatically elevated [1–4]. At all phase of COVID-19, a surge in mental health problems (e.g., depression and anxiety) were observed among various population such as infected people, healthcare workers, and the general population [5–14]. For instance, a meta-analysis including 66 studies indicated that during the COVID-19 pandemic, the pooled prevalence of depression among infected individuals, healthcare workers, and the general population was 41.7%, 31.0%, and 31.5%, respectively; the pooled prevalence of anxiety among these population was 42.3%, 29.8%, and 29.0%, respectively [8]. Another meta-analysis involving 31 studies demonstrated that the pooled prevalence of depression and anxiety among COVID-19 infected people was 45% and 47%, respectively [15]. Examples of factors associated with depression and anxiety among general population included worry about infection [16], uncertainties [17, 18], and negative perceptions toward the pandemic [19]. There are reasons to believe that the prevalence of depression and anxiety would be high in the end phase of COVID-19 pandemic in China.

### Perceived risk and depression/anxiety

The perception of potential threats and risks may affect individual’s emotional response such as depression and anxiety. Perceived risk, i.e., individual’s perception and awareness of objective risks existing in the outside world [20], emphasizes the influence of the individual’s experience gained from intuitive judgment and subjective perception on the individual’s cognition [20, 21]. Individual’s subjective interpretations of risk could affect their behavior and emotional responses when facing new, unobservable, and unpredictable hazard such as COVID-19 [22]. Li & Lyu reported that higher perceived risk was associated with poorer mental health outcomes during the acute phase of COVID-19 pandemic [21]. Perceived COVID-19 infection risk was positively associated with depressive symptoms in young adults in quarantine [23] and in healthcare workers [24]. Additionally, Alsolais et al. reported that higher level of self-reported perceived risk was associated with greater depressive symptoms and anxiety in their longitudinal study [25]. Similar results have been found in other infectious diseases, such as SARS and Ebola virus [26, 27].

### Potential mediating role of fear of COVID-19

It is well-established that cognition and emotion regarding a specific disease are associated with mental distress [28]. Fear, characterized as an unpleasant emotion [29], could be harmful to mental health [30]. Previous study revealed that fears of COVID-19 was positively associated with perceived risk during the pandemic [31], and also with depression and anxiety [32]. A recent study suggested that fear of COVID-19 could potentially mediate the relationship between perceived risk and mental distress among healthcare workers [33]. The associations can be explained by emotional illness representation of a disease, i.e., how people feel about the disease [34, 35]. Thus, it is plausible that COVID-19 perceived risk would increase fear of COVID-19, which would in turn increase depression and anxiety, i.e., fear of COVID-19 may be a mediator between COVID-19 perceived risk and depression/anxiety. The potential associations imply that reducing fear of COVID-19 may lessen the high prevalence or reduce severity of mental distress among the public.

### Potential moderating role of resilience

Resilience is a dynamic process by which individuals adapt well to stressful events [36]. In this process, individuals make full use of their personal and psychological resources to effectively cope with negative life events such as stress, frustration and trauma as well as to survive adversity [36, 37]. The importance of resilience stems from its ability to help individuals actively adapt to severe adversity, maintain mental health, recover from disasters [38] and protect individuals from depression [39] and anxiety [40]. Thus, individuals who experienced depressive symptoms and anxiety could benefit from a high level of resilience [40, 41]. The importance of resilience is also highlighted at various phases of the pandemic [42, 43]. For example, that the higher COVID-19-related resilience, the less severe depressive symptoms was observed in a group of healthcare workers [44]. Therefore, it is plausible to hypothesize that resilience might reduce the negative consequences of fear of COVID-19 on depression/anxiety, i.e., resilience may weaken the associations between fear of COVID-19 and depression/anxiety.

### Objective of the present study

The present study was conducted among the general population in Wenzhou, China from January 11, 2023 to January 29, 2023, right after the lifting of almost all control measures. COVID-19 prevention and control measures in China changed so suddenly from extremely high to none in a very short time so that most people were unprepared. Given that background, this study investigated (1) the prevalence of depression and anxiety; (2) the associations among COVID-19 perceived risk, fear of COVID-19, resilience, depression, and anxiety; (3) the mediating role of fear of COVID-19 between COVID-19 perceived risk and depression/anxiety; (4) the moderating role of resilience between fear of COVID-19 and depression/anxiety (See Supplementary Fig. 1).

## Methods

### Participants and data collection

Data were collected through an online survey among a convenient sample of 947 participants aged 18–60 in Wenzhou, China from January 11 to 29, 2023, via Wenjuanxing which is a widely used professional online survey platform in China. Participants were informed that participation in the survey was voluntary and anonymous and they were not required to provide any personal information. All information collected would be used for research only and kept strictly confidential. Once informed of the above, participants could choose to participate in or withdraw from the survey. The time required to complete the survey was approximately 10 min.

To ensure timeliness of the study, convenience sampling was used as people’ s mental distress, fear, and perceived risk may change quickly as the pandemic moved through its stages. Data from 947 participants were collected, of which 12 were excluded because they either took less than 4 min (mean completion time = 11.57 min, SD = 7.50 min) to complete the survey or showed response set in the answer. Finally, data obtained from 935 was analyzed.

### Measurements

#### Background factors

Background information was collected including sex, age, educational level, religious belief status, family economic status, whether having medical background, marriage status, and physical status.

#### Depression

The 9-item Patient Health Questionnaire (PHQ-9) was used to evaluate depression [45]. It was rated on four-point Likert scales (0 = not at all to 3 = nearly every day), with total scores ranging from 0 to 27. Summative scores of 0–4, 5–9, 10–14,15–19, 20–27 indicate no depression, mild depression, moderate depression, moderately severe depression, and severe depression, respectively. Its Chinese version has been validated and showed excellent psychometric properties in previous studies [5, 46]. The Cronbach’s alpha of the scale was 0.92 in this study.

#### Anxiety

Generalized Anxiety Disorder-7 (GAD-7) was used to evaluate anxiety [47]. It is a seven-item scale rated on four-point Likert scales (0 = not at all to 3 = near every day), with total scores ranging from 0 to 21. The summative scores of 0–4, 5–9, 10–14,15–21 reflect no anxiety, mild anxiety, moderate anxiety, and severe anxiety. Its Chinese version has been validated and showed excellent psychometric properties in previous studies [7, 46]. The Cronbach’s alpha of the scale was 0.95 in this study.

#### COVID-19 perceived risk

The COVID-19 Risk Perception Scale was developed to assess COVID-19 perceived risk in Chinese population [48]. It consists of 9 items including three dimensions (perceived susceptibility, severity, and controllability of COVID-19) rated on a 5-point Likert scale (1 = negligible to 5 = very large), with a total score ranging from 9 to 45. The Cronbach’s alpha of the scale was 0.90 in this study.

#### Fear of COVID-19

The fear of COVID-19 was evaluated by using the fear of COVID-19 Scale, which is reliable and validated in assessing the fear of COVID-19 among the general population [49]. It consists of 7 items rated on a 5-point Likert scale (1 = strongly disagree to 5 = strongly agree), with a summative score ranging from 7 to 35. Its effectiveness and superiority for assessing fear of COVID-19 were indicated in Chinese population [50–54]. The Cronbach’s alpha of the scale was 0.93 in this study.

#### Resilience

Resilience was assessed using a short version of the Connor-Davidson Resilience Scale (CD-RISC). It reflects the ability to tolerate experiences, such as change, personal problems, illness, pressure, failure, and painful feeling [55]. Its Chinese version was demonstrated good reliability and validity [56, 57]. It is a 10-item scale rated on a 5-point Likert scale (0 = not true at all to 4 = true nearly all of the time), with total scores ranging from 0 to 40. The Cronbach’s alpha of the scale was 0.97 in this study.

### Statistical analysis

Descriptive analyses were conducted for background factors and the prevalence of depression and anxiety. Simple linear regression analyses were performed to test the associations between background factors and depression (PHQ-9 score)/anxiety (GAD-7 score). Pearson correlation coefficients were derived to test associations between variables, i.e., COVID-19 perceived risk, fear of COVID-19, resilience, PHQ-9 for depression, and GAD-7 for anxiety. Structural Equation Modeling (SEM) with Maximum Likelihood (ML) estimator and adjusted for significant background factors was performed to test the moderated mediation. COVID-19 perceived risk was constructed as a latent variable, while other variables were observed variables. Satisfactory model fit indices included Comparative Fit Index (CFI ≥ 0.90), Tucker-Lewis Index (TLI ≥ 0.90), Root Mean Square Error of Approximation (RMSEA ≤ 0.08), and Standardized Root Mean Square Residual (SRMR ≤ 0.08) [58]. Moderated mediation was tested using Mplus 7.3 and other analyses were done with SPSS 23.0. Statistical significance was defined as two-tailed *p*-value < 0.05.

## Results

### Descriptive statistics

As shown in Table 1, the majority of the sample were female (79.3%), aged over 40 (52.1%), currently married (90.3%), had a college or above degree (42.2%), had no religious belief (66.5%), had very poor/poor family economic status (58.4%), had no medical background (88.2%), or had healthy physical status (92.1%). Of all participants, 23.7% exhibited moderate to severe depression (PHQ-9 ≥ 10) and 9.5% with moderate to severe anxiety (GAD-7 ≥ 10).

**Table 1:** Descriptive statistics and background factors of depression and anxiety

	n (%)	Depression (PHQ-9 score)		Anxiety (GAD-7 score)	
		Mean (SD)	β (SE)	Mean (SD)	β (SE)
**Sex**					
Male	194 (20.7)	6.24 (5.65)	Reference	3.80 (4.41)	Reference
Female	741 (79.3)	6.92 (5.66)	0.05 (0.46)	4.06 (4.57)	0.02 (0.36)
**Age**					
≤ 40	448 (47.9)	7.69 (5.08)	Reference	4.63 (4.59)	Reference
> 40	487 (52.1)	4.38 (4.73)	-0.10** (0.42)	2.32 (4.08)	-0.09** (0.34)
**Educational level**					
Middle school or below	251 (26.8)	6.10 (5.66)	Reference	3.52 (4.12)	Reference
High school or equal	289 (30.9)	6.85 (5.74)	0.06 (0.49)	3.94 (4.70)	0.04 (0.39)
College or above	395 (42.2)	7.14 (5.58)	0.09* (0.46)	4.35 (4.65)	0.09* (0.37)
**Religious belief status**					
Yes	313 (33.5)	6.70 (5.79)	Reference	3.92 (4.42)	Reference
No	622 (66.5)	6.82 (5.61)	0.01 (0.39)	4.04 (4.60)	0.01 (0.32)
**Family economic status**					
Very poor/poor	546 (58.4)	6.97 (5.67)	Reference	4.14 (4.43)	Reference
Average	356 (8.1)	5.56 (5.62)	-0.04 (0.39)	3.82 (4.67)	-0.03 (0.31)
Good/very good	33 (3.5)	5.88 (5.98)	-0.04 (0.40)	3.73 (4.86)	-0.02 (0.81)
**Medical background**					
Yes	110 (11.8)	7.85 (5.80)	Reference	4.62 (4.88)	Reference
No	825 (88.2)	6.63 (5.63)	-0.07* (0.57)	3.92 (4.49)	-0.05 (0.46)
**Marriage status**					
Currently married	844 (90.3)	6.65 (5.68)	Reference	3.89 (4.47)	Reference
Others (unmarried, divorce, separation)	91 (9.7)	7.97 (5.43)	0.07* (0.62)	5.27 (5.10)	0.08* (0.50)
**Physical status**					
Healthy	861 (92.1)	6.68 (5.66)	Reference	3.89 (4.47)	Reference
Others (having chronic disease, pregnancy, having serious illness)	74 (7.9)	7.93 (5.64)	0.10* (0.69)	5.27 (5.10)	0.08* (0.50)
**Depression**					
No	365 (39.1)				
Mild to moderate (PHQ-9 = 5–9)	348 (37.2)				
Moderate to severe (PHQ-9 ≥ 10)	222 (23.7)				
**Anxiety**					
No	560 (59.9)				
Mild to moderate (GAD-7 = 5–9)	286 (30.6)				
Moderate to severe (GAD-7 ≥ 10)	89 (9.5)				

*p < 0.05; **p < 0.01

The mean [standard deviation (SD)] values were 6.77 (SD = 5.66) for PHQ-9, 4.00 (SD = 4.54) for GAD-7, 26.73 (SD = 6.40) for COVID-19 perceived risk, 19.15 (SD = 5.77) for fear of COVID-19, and 24.75 (SD = 9.36) for resilience (Table 2).

**Table 2:** Pearson correlation analysis

	Mean, SD (range)	1	2	3	4	5
COVID-19 Perceived risk	26.73, 6.40(9–45)	1				
Fear of COVID-19	19.15, 5.77(7–35)	0.47**	1			
Resilience	24.75, 9.36(0–40)	-0.03	-0.14**	1		
Depression	6.77, 5.66(0–27)	0.37**	0.39**	-0.10**	1	
Anxiety	4.00, 4.54 (0–21)	0.32**	0.45**	-0.09**	0.82**	1

**p < 0.01.

### Background factors of depression and anxiety

Participants aged over 40, or were currently married, or were healthy were less likely to have depression or anxiety. Those who had medical background were more likely to have depression but not anxiety. Depression and anxiety was higher among those with a college or above degree. No significant differences were found across sex, religious belief status, and family economic status groups (Table 1).

### Pearson correlation analysis

As shown in Table 2, COVID-19 perceived risk, fear of COVID-19, depression, and anxiety were significantly and positively associated with each other (*r* between 0.32 and 0.82). Depression (*r* = -0.10), anxiety (*r* = -0.09) and fear of COVID-19 (*r* = -0.14) were significantly and negatively associated with resilience. The correlation between COVID-19 perceived risk and resilience was statistically non-significant.

### Results of moderated mediation

**Fig. 1:**
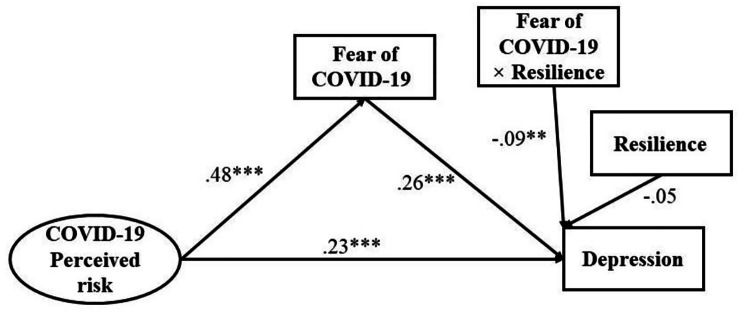
Structual equation modeling for Model 1. Standardized coefficients were reported. **p < 0.01; ***p < 0.001. The model was adjusted for age, educational level, medical background, marriage status, and physical status

**Fig. 2:**
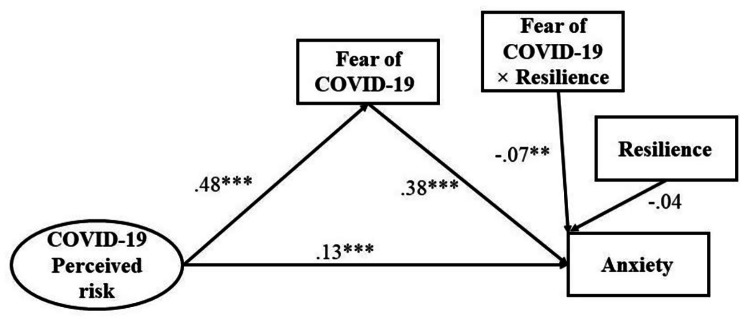
Structual equation modeling for Model 2. Standardized coefficients were reported. **p < 0.01; ***p < 0.001. The model was adjusted for age, educational level, marriage status, and physical status

Depression and anxiety were used as two outcome variables. Thus, two models were generated (Model 1: depression and Model 2: anxiety), which were adjusted by significant background factors (Table 3, Fig. 1**and** Fig. 2). Significant background factors associated with depression were age, educational level, medical background, marriage status, and physical status, while significant background factors associated with anxiety were age, educational level, marriage status, and physical status (Table 1). Both models showed satisfactory fit indices (Model 1: χ^2^/*df* = 2.33, CFI = 0.97, TLI = 0.96, SRMR = 0.04, RMSEA = 0.04; Model 2: χ^2^/*df* = 2.40, CFI = 0.96, TLI = 0.95, SRMR = 0.04, RMSEA = 0.05).

**Table 3:** Results of moderated mediation

	Model 1		Model 2	
	Fear of COVID-19	Depression	Fear of COVID-19	Anxiety
	*β*(SE)	*β*(SE)	*β*(SE)	*β*(SE)
COVID-19 perceived risk	0.48***(0.03)	0.23***(0.03)	0.48***(0.03)	0.13***(0.03)
Fear of COVID-19		0.26***(0.03)		0.38***(0.03)
Resilience		-0.05(0.03)		-0.04(0.03)
Fear of COVID-19 × Resilience		-0.09**(0.03)		-0.07**(0.02)

Model 1 was adjusted for age, educational level, medical background, marriage status, and physical status. Model 2 was adjusted for age, educational level, marriage status, and physical status. **p < 0.01; ***p < 0.001

In Model 1, fear of COVID-19 significantly mediated the relationship between COVID-19 perceived risk and depression. COVID-19 perceived risk was positively associated with fear of COVID-19 (*β =* 0.48, SE = 0.03, *p* < 0.001), which was in turn positively associated with depression (*β =* 0.26, SE = 0.03, *p* < 0.001). As the direct effect of COVID-19 perceived risk on depression was statistically significant (*β =* 0.23, SE = 0.03, *p* < 0.001), a partial mediation effect was observed with a mediation effect size of 35.17% (*p* < 0.001). In Model 2, fear of COVID-19 significantly mediated the association between COVID-19 perceived risk and anxiety, i.e., COVID-19 perceived risk was positively associated with fear of COVID-19 (*β =* 0.48, SE = 0.03, *p* < 0.001) which in turn was positively associated with anxiety (*β =* 0.38, SE = 0.03, *p* < 0.001). The direct effect of COVID-19 perceived risk on anxiety was also statistically significant (*β =* 0.13, SE = 0.03, *p* < 0.001) and the partial mediation effect with a mediation effect size was 14.10% (*p* < 0.001).

**Fig. 3:**
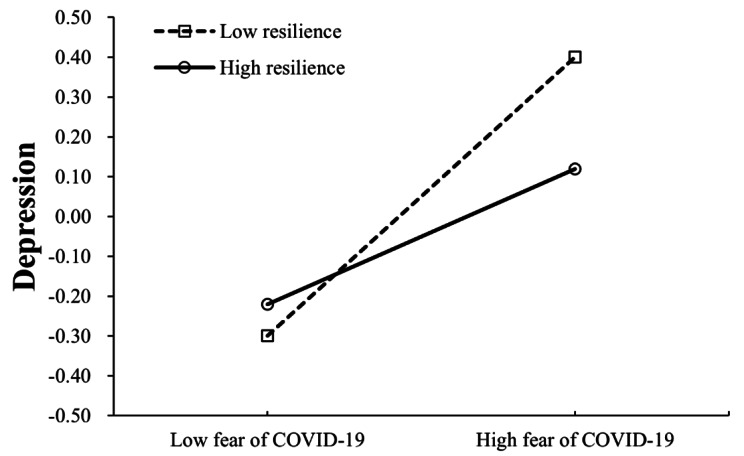
Interactive effect of resilience and fear of COVID-19 on depression (standardized)

**Fig. 4:**
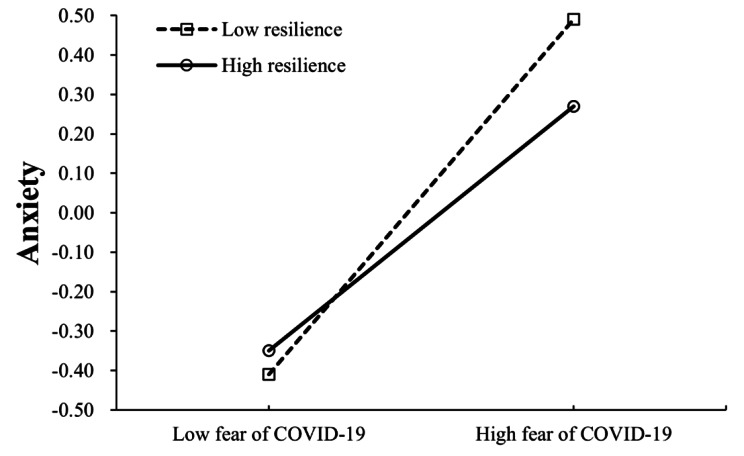
Interactive effect of resilience and fear of COVID-19 on anxiety (standardized)

Moderating effect of resilience on the relationship between fear of COVID-19 and depression/anxiety was supported in that the relationships become weaker when resilience was high. Variables were centralized prior to creating their product terms and standardized score of depression or anxiety was applied. The results showed that the interaction term of resilience and fear of COVID-19 was negatively related to depression (*β* = -0.09, SE = 0.03, *p* < 0.01; Model 1). Simple slopes analysis was conducted to further interpret the results. The interaction plot in Fig. 3 showed that the positive association between fear of COVID-19 and depression was stronger in participants with low resilience (1 SD below the mean, simple slope: *β*_*Low*_ = 0.35, SE = 0.04, *p* < 0.001) than that in participants with high resilience (1 SD above the mean, simple slope *β*_*High*_ = 0.17, SE = 0.05, *p* < 0.001). It is also indicated that interaction term of resilience and fear of COVID-19 was negatively related to anxiety (*β* = -0.07, SE = 0.02, *p* < 0.01; Model 2). The interaction plot in Fig. 4 showed that the association between fear of COVID-19 and anxiety was stronger in participants with low resilience (1 SD below the mean, simple slope: *β*_*Low*_ = 0.45, SE = 0.03, *p* < 0.001) than that in participants with high resilience (1 SD above the mean, simple slope *β*_*High*_ = 0.31, SE = 0.04, *p* < 0.001).

## Discussion

This timely study was conducted during the end phase of the COVID-19 pandemic in China from January 11 to 29, 2023. At the end of 2022, almost all measures were lifted in mainland China. Before lifting all the measures, China’s infection rate was less than 1% [59], indicating very low exposure of the public to COVID-19 infection. The abrupt relaxation of prevention and control policies exposed a majority of Chinese population to the infection [60]. Rapid policy shifts and exposure to infections might challenge public trust in the health care system and authorities, which may in turn affect their mental health and increase depression and anxiety [61]. Furthermore, the public was unprepared for the sudden changes and China also faced a shortage of medical resources. In such situations, unpreparedness and increasing demand for medical supplies made obtaining treatment difficult, which subsequently contributed to the rise of mental distress [60]. It reminded us that public trust and preparedness are important to maintain mental health [61]. Trust fostered a sense of security, which helped to reduce mental distress. Preparedness empowered individuals to cope with challenges and mitigated the psychological toll of unexpected circumstances.

Our study found high prevalence of moderate to severe depression (PHQ-9 ≥ 10 = 23.7%) and prevalence of moderate to severe anxiety (GAD-7 ≥ 10 = 9.5%) in Chinese adult general population. This finding was consistent with previous research conducted during the acute phase of the COVID-19 pandemic in Wuhan, with 20.3% depression rate using the same instruments and cut-off value [46]. However, the prevalence of moderate to severe anxiety in our study was much lower than the reported anxiety rate of 18.9% in Wuhan [46]. Variations may be explained by numerous factors such as the differences in the features of the different pandemic phases and participants’ characteristics [62]. Despite variations, our results indicated that people at the end phase of COVID-19 still had high levels of mental distress.

In line with published literature, those aged over 40 or unmarried, divorced or separated, or with chronic disease, pregnancy, serious illness were more likely to have depression and anxiety [63, 64]. Interestingly, sex and religious belief status were not significant factors. Consistent with finding of a meta-analysis [15], no significant sex differences was found in the prevalence of depression and anxiety. Possible reason was that during such special period, males and females were equally mentally-distressed. Religious belief was found as a protective factor against mental health problems in COVID-19 pandemic in previous study [65, 66], but not in the current study. Some of the background factors seemed non-significant in affecting depression and anxiety in the Chinese general population.

The present study revealed significantly positive associations among COVID-19 perceived risk, fear of COVID-19, depression and anxiety. This suggested that public’s risk perception towards COVID-19 and the fear of COVID-19 deserved more attention on account of their roles in mitigating depression and anxiety. Similar positive associations were found among Saudi nursing students during the COVID-19 pandemic [25]. The results showed that COVID-19 perceived risk had positive direct effect on depression and anxiety, which was consistent with previous findings on epidemic risk perception and mental health [21, 24]. The emergence of epidemic created lots of uncertainty, posed threats to individuals and sharply increased individuals’ perceived risks, which resulted in anxiety and depression [67]. People also experienced loss of control and powerlessness during great pandemic and felt that they could only wait passively through the development of the epidemic, which brought to them higher levels of depression and anxiety [21].

One of the key findings was that fear of COVID-19 partially mediated the associations between COVID-19 perceived risk and depression/anxiety. It supported the hypotheses that fear of COVID-19 was both associated with COVID-19 perceived risk and depression/anxiety, which was partially in line with the Common Sense Model (CSM), i.e. an illness determines emotional responses that further affects mental health [34, 35]. Also, Beck’s cognitive theory proposed that cognitive content (including individuals’ belief systems, expectations, assumptions, and evaluations) is activated by events and driven by subjective meaning that interacts with their affective systems [68]. Therefore, exaggerated interpretations of threats, including fear of COVID-19 [69], may lead to inappropriate or excessive anxiety and depression [70]. Yıldırım et al.’s finding supported the mediation role of coronavirus fear between coronavirus risk and parental coronavirus anxiety among healthcare workers [71]. Notably, in the current study, the mediation effect size was 35.17% for depression and 14.10% for anxiety. It suggested that the decrease in COVID-19 fear could reduce depression and anxiety. As individual’s COVID-19 perceived risk and fear of COVID-19 could change over time, future longitudinal studies would provide valuable insights to the dynamics among perceived risk and fear and mental distress and also discover other potential mediators.

Another key finding was that resilience weakened the impact of fear of COVID-19 on depression/anxiety. Consistent with previous findings, high resilience was a protective factor against depression and anxiety [72]. Individuals with higher resilience may be more positive and more likely to use active coping strategies which help reduce the impact of fear on how much depression/anxiety they develop [73]. More resilient people may also possess more effective emotion regulation skills to weaken the effect of fear on depression and anxiety, despite the existence of subjective fear of COVID-19 [74]. Zhou et al. showed that resilience moderated the association between fear and depression among middle school students after earthquake [73]. Besides resilience, which has been widely studied as a positive psychological resource, research could also examine other potential moderators such as self-compassion, which is a caring, nonjudgmental lens in the face of personal suffering [75] and is another resistance factor for mental health problems [76].

In sum, the present study supported the hypotheses and had great implications. Firstly, it facilitated a better understanding of how COVID-19 perceived risk resulted in depression and anxiety. It thus provided theoretical guidance for future epidemic intervention [21]. Secondly, an important reminder is that fear of COVID-19 has emotional components. Partially according to CSM, emotional components would affect mental health [34, 35]. To reduce depression and anxiety among the public, interventions are needed for such components. Fear of COVID-19 was an important mediation mechanism by which the perceived risk of unexpected epidemic affected individuals’ mental health. Coping with the fear of COVID-19 was an important means to reduce mental distress during such crisis. It is well-known that fear stems from uncertainties and the unknown. Thus, intervention and measures need to focus on helping individuals gain more knowledge about COVID-19 and increase availability of drug and treatment. Thirdly, the mediation and moderation relationship found in the current study allow us to explore various ways to attenuate mental distress in pandemics, rather than taking a single approach. For example, as resilience is a resistance factor to mental health problems, especially in pandemics, efforts could be directed to increase public’s resilience, through actions via well-suited online resilience-based interventions or adding psychological counseling and mental health services [77, 78].

Despite its strengths, this study has some limitations. Firstly, social desirability bias could exist. For instance, fear of COVID-19, depression and anxiety may be underreported due to potential stigma and discrimination [79]. Secondly, this study failed to calculate the participation rate. Thirdly, given the feature of cross-sectional design, no causal inferences can be made. Future longitudinal studies are needed to verify the such associations as COVID-19 perceived risk and fear of COVID-19 would change over time. Fourthly, the distribution of participants characteristics was not representative of the Chinese population as convenience sampling was used. For instance, the majority of our participants was female (79.3%). Cautions are warranted to generalize the results to the entire Chinese population and to other countries. However, through adjusting the models with demographic variables the current study aimed to maximize its generalizability.

## Conclusion

The present study detected high prevalence of moderated to severe depression (23.7%) and relatively low prevalence of moderate to severe anxiety (9.5%) among Chinese adult population after lifting all measures. The associations between COVID-19 perceived risk and depression/anxiety were partially mediated via fear of COVID-19. Resilience moderated the relationships between fear of COVID-19 and depression/anxiety. Due to vaccination uptake and less virulent variants, it seemed that the COVID virus became weaker and more controllable, caused fewer and less severe symptoms, and induced fewer emotional responses over the last three years till the end of 2022. Yet with sudden lifting all measures in January, 2023, panic and mental distress surged. Depression and anxiety caused by fear of COVID-19 needs to be highlighted. It is important to reduce people’s perceived risk towards COVID-19 to decrease the fear of COVID-19, which in turn reduces depression and anxiety. Interventions and efforts are necessary to enhance resilience of the public when epidemic crisis emerged.

### Electronic supplementary material

Below is the link to the electronic supplementary material.

Supplementary Material 1

## Data Availability

The datasets used and/or analyzed during the current study are available from the corresponding authors on reasonable request.

## Abbreviations

COVID-19: Corona Virus Disease 2019
SARS: Severe acute respiratory syndrome
CSM: Common Sense Model
PHQ-9: The 9-item Patient Health Questionnaire
GAD-7: Generalized Anxiety Disorder-7
SEM: Structural Equation Modeling
CFI: Comparative Fit Index
TLI: Tucker-Lewis Index
RMSEA: Root Mean Square Error of Approximation
SRMR: Standardized Root Mean Square Residual
SPSS: Statistical package for social sciences
SD: Standard deviation
SE: Standard error
