# Supplementary figures and images for: Perceived risk of COVID-19 hurts mental health: the mediating role of fear of COVID-19 and the moderating role of resilience
Source: BMC Psychiatry. 2024 Jan 22;24:58. doi: 10.1186/s12888-024-05511-x (PMC10802027; doi:10.1186/s12888-024-05511-x)

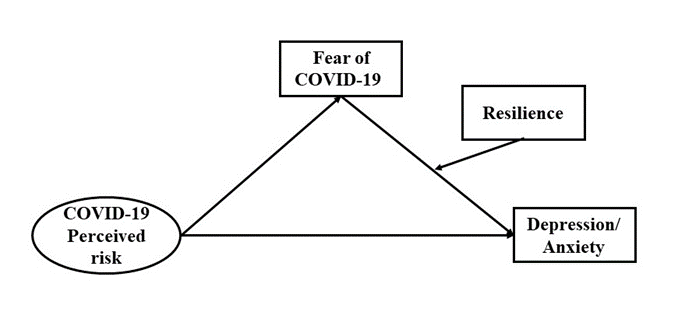


Fig. 1 The hypothesized moderated mediation model

Supplement: Supplementary file 1 — Supplementary Material 1 [file 12888_2024_5511_MOESM1_ESM.docx]
